# Book Reviews

**Published:** 1809-03

**Authors:** 


### CRITICAL ANALYSIS
OF THE
### RECENT PUBLICATIONS
ON THE
### DIFFERENT BRANCHES OF PHYSIC, SURGERY, AND MEDICAL PHILOSOPHY.

*A Letter to* John Haygarth, M. D. F. R. S. *London and Edinburgh, &c. from* Colin Chisholm, M. D. F. R. S. &c. *Author of an Essay on Pestilential Fever, exhibiting further Evidence of the infectious Nature of this fatal Distemper in Grenada, during the Years* 1794, 5, and 6, *and in the United States, of America, from* 1793 to 1805, *in order to Correct the pernicious Doctrine promulgated by Dr.* Edward Miller, *and other American Physicians, relative to this destructive Pestilence.* 8vo. pp. 272. London.

Though there is not a more important inquiry than concerning the laws of infection, yet the subject seems to have been fairly handled by very few indeed. Dr. Chisholm has always been a steady supporter of the contagious property of this disease; and to do him justice, he has been more precise in his statement of facts and consequent inferences, in proportion as he has been called upon by the opposition of others; on this occasion he brings the aid of various, and, we doubt not, impartial authorities, in support of his own.

A considerable part of the work before us is taken up with references to the Author's "Essays." To the New-York, and other American periodical separate publications. To "the Collection of Facts" preserved by the industrious Mr. Webster; and to many other records both in print and MS. These are numerous, and many of them stated with so much attention to brevity, that it is impossible we should offer any abstract of them, which could be at the same time correct and consistent with our limits; we shall therefore confine ourselves principally to some original papers. The first of these is the following letter from Dr. M'Gregor.

*Portsmouth, 3d Jan.* 1808.

Sir,

"Without any communication of views or of opinions, I find that I have been concurring with you in a very important and much disputed point, viz. the contagious nature of fever in the West Indies. I myself entertain no doubt that the fever which I saw in Grenada, in the year 1796, and which proved so destructive to the army there, was communicated by contagion. I understand contagion in the sense in which Dr. Adams explains it in the second edition edition of his work on Morbid Poisons. The strong circumstances which led me to conclude that the fever which I saw at Grenada and Tortola was contagious, I have detailed in the Appendix to the Sketches of the expedition from India to Egypt. A very recent reference to my notes and case books, written in the West Indies, confirms me in the opinion which I had formed of this fever. I have likewise lately corresponded with my friend Mr. Bruce, surgeon, 88th regiment, on this subject. Mr. Bruce is well qualified to speak on the subject, he was my assistant in the 88th regiment, and was with me in the Betsy transport, at Grenada and Tortola. In his letter, Mr. Bruce offers some remarks and corrections of the account of the fever, which I have appended to the Sketches. He says, that 'the fever which prevailed among the 88th regiment, in the Betsy transport, in the West Indies in the year 1796, was manifestly contagious and very fatal. A large proportion of the officers of other regiments, particularly of the 8th, were ill of it at the same time,' &c. By a singular coincidence, which I think forms the strongest corroboration of your statement, without the most distant knowledge of me or my opinions, occurs in p. 314, of the second volume, second edition of your work, in which you allude to the melancholy situation which Mr. Bruce and myself witnessed at Tortola, and of which I have given some account in the Appendix to my Sketches

I am, &c.

"JAMES M'GREGOR."

Dr. Blane's letter to the Prussian minister, which we have noticed in a former Number, is next attended to, with Dr. Fellows's account of the Gibraltar fever, and also the fever on board the Hankey, which is supposed to have conveyed contagion from Africa to the West Indies.

The next "Proposition" of our author is the most important, and has been too little attended to, viz. That this fever and the yellow remittent fever, are not "precisely the same disease." How far they may resemble each other in character cannot be exactly ascertained amidst the various descriptions we have received of individual cases from so many quarters; but it is a most important inference, which we shall constantly keep in view, that the yellow remittent fever may not be contagious, though another fever, said to have been imported, may come within that description. Now, no one will dispute that an army, or ship's crew, arriving in a diseased state, may prove infectious to those who have communication with them. Whether this is all that the ship Hankey can be accused of, may be in some measure guessed by the opening of the succeeding proposition.

PROPOSITION

"PROPOSITION III.

"*That my deduction of the disease from the pestilential state of the ship Hankey is just, correct, and supported by evidence corroborative of that which I received from Mr. Paiba.*

"The result of a very attentive consideration of the circumstances of the ship Hankey, as they were represented to me, on the arrival of that ship at Grenada, I have already stated to be, '*that a fever, proceeding originally, perhaps, from the inclemency of the season, and the circumstances of the situation of the adventurers, had become by confinement, filth, consequent impurity of air, and depression of spirits, a true jail fever, or a fever of infection heightened to an almost pestilential violence.*' Making this then my 'orationis argumentum,' I shall proceed to the establishment of my third proposition. In the course of my investigation for this purpose, we shall find that all difference between my statement of the situation of the Hankey, and that described by the more intelligent colonists of Bulama, exists in the expression of, not in the facts themselves."

The following conclusion, we confess, does not appear to us perfectly reconcilable with the opening.

"To conclude," says Dr. Chilsholm, "let the unbiassed reader now become umpire, and decide whether or not 'a nova pestis—a peculiar, original, foreign pestilence, recently generated, and utterly unknown before, endued with a new and distinct character, possessing new powers of devastation, and capable of propagating itself by contagion throughout the world,' was introduced by the Hankey on the 19th February, 1793, into Grenada. The demonstration of my senses, and the clearest perception of my mind, assure me it was a nova pestis, to which the observation of Thucydides is truly applicable; for never before had so dreadful a pestilence occurred in the West Indies, nor such destruction of the human race been recorded; οὐ μεντοι τοσουτος γε λοιμος, οὐδε φθορά οὕτω ανθρωπων ουδαμου εμνημονευετο γενεσθαι. Indeed, if we make a parallel between the two fevers, that of Grenada and the τοσουτος γε λοιμος of Athens, we shall find that they bear a strong affinity to each other in their causes and symptoms. They seem indeed to have been the same disease, the same 'destructive monster.' If they differ in one symptom, the large bilious discharges noticed in the Athenian αποκαθαρσεις χολῆς, they agree in so many other circumstances as almost to establish their identity."

We shall presently see, that whatever novelty of symptoms may have occurred to distinguish this from the camp fever, are afterwards accounted for by Dr. Chilsholm; but by the lines above quoted, we are at some loss to know whether our author would be understood to mean that this contagion remained concealed somewhere in Africa, from the time of Thucidydes, till it appeared in Grenada. A great deal of learning follows, to prove that the pestilence of Athens was a contagion introduced by commerce. This is a question we shall not dispute, but it is certain that the crowded and distressed state of the city at that time was sufficient to induce camp fever among the inhabitants. It is however much more to our purpose to enquire into those facts which are not at present within our reach, and which daily experience may correct.

The object of the Fourth Proposition is to show, that "the author's letter, published in a mutilated form in the Medical Repository of New York, and quoted by Dr. Miller, he [Dr. Chisholm,] has not relinquished the doctrine and opinion upheld in his Essay.

"It is wonderful," "says our author," it is almost beyond belief, that ingenious and well-informed men, (I mean Drs. Mitchell and Miller, of New York) should endeavour to impress on the minds of their readers, that I have rescinded this opinion; that they should artfully insinuate, by a very singular perversion of a very clear and unelaborate statement of facts, that I have coalesced with themselves. The fact is, that the fever generated on board the ship Marie Anne, in her voyage from Liverpool to Surinam, and thence to Demerary, was absolutely infectious; but its contagion, *sublimed* by climate and uniting probably with the marsh miasms which abounded at the time in the neglected trenches and canals of Stabroek, produced a fever of a mixed type, 'the destructive monster' of the Hankey. It is evident that this has been uniformly my opinion since 1793; so that when Drs. Mitchell and Miller concede that the fever of North America, since that year, has not been uniformly endemic, a prospect will then be held out of union and coalescence of sentiment; but at present our opinions are as two lines which approximate but cannot meet: what these ingenious gentlemen are to yield, is what they now profess to dissent from. 'But in two points we dissent from Dr. Chisholm: first, in his opinion of the *peculiar nature* of the pestilential disease which appeared in Grenada and Demerary, and of its differing essentially from the yellow fever as it prevails in the West Indies and on the American continent. And, secondly, in his opinion of the contageousness of the pestilential disease generated on board of ships, while he justly acknowledges that the yellow fever is not capable of being propagated by such means.' Med. Repository, v. 5, p. 233. I am rejoiced to see that they have manifested an inclination to this necessary concession; a concession which is in truth the sober dictate of common sense; a concession which would do equal honour to their judgment and their humanity; a concession which would remove all obstacles to personal and commercial safety; a concession which every circumstance of the case most obviously requires. Ibid. p. 231. 233. The compound which results from the state of circumstances these gentlemen detail, p. 233, I have above adverted to, and they have virtually admitted, although they endeavour to evade the effect of this admission, by a concluding dissentient remark: 'As to the mixed or hybrid forms of malignant fever, concerning which Dr. Chisholm offers his conjectures, we can only observe, that they appear to us to be founded upon an analogy analogy too remote and fanciful to serve as the basis of sound and correct reasoning.' This may be considered as the still smoking but nearly extinguished embers of controversy; and, for the benefit of mankind, may its obsequies be duly solemnized.

"It would have been well that Dr. Mitchell and Dr. Miller had adverted to a fact which I communicated to my correspondent in the letter which contains the subject of our present discussion. They would probably join me in execrating the authors of the calamity; in saying with me, that 'Captain Cox, of the Hankey, and Captain M'Nab, of the Marie Anne, merit the most exemplary punishments.'—'Aug. 30th. Having had no opportunity by which I could forward this letter, I shall fill the remainder of the sheet with an account of a remarkable instance of infection on this coast, (east sea coast of Demerary). A young man, of the name of Allison, arrived only in June last from England, his native country, was unfortunately exposed in town to the contagion of the fatal fever, which I have given a general history of. His brother, anxious to remove him from a pestilential air, went to town; and, although the young man was already labouring under the incipient symptoms of the pestilential remittent, yet he insisted on his proceeding up the east coast. On the 26th or 27th of July they stopped for the night at Lusignan, the plantation of K. F. Mackenzie, Esq. three miles further from town than Success, and consequently eleven together. They put up with the manager, Mr. Reid, who, from mere humanity, gave up his hammock to the sick man. On the following morning, the two brothers continued their journey to windward, and that evening the sick man expired. The manager of Lusignan, having no suspicion of the nature of Allison's fever, took no precaution to destroy the seminia of infection retained by the hammock, but made use of it as he had been wont do. An overseer and two white carpenters, natives of Scotland, lodged in the same house. The former and one of the latter, being probably more disposed to be acted on by the infection, were the first who suffered by it: the overseer died on the seventh day, the carpenter recovered with difficulty. Mr. Reid was not affected for a considerable time; at length, about the middle of this month (August), he fatigued himself in the course of his duty, and instantly a predisposition was formed. This is an instance of accumulation of morbid agency, inactive till put in motion by a stimulus, like the electric spark. Similar instances were frequently observed both at Grenada and Philadelphia during the presence of pestilence at these places. On the evening of the 23rd, he was attacked with a fever, the description of which seems, from Mr. Mackenzie's and Dr. Ord's statement, to have been for two days remittent. On the third day, however, its type, and the aspect of the patient, became altogether different, and decidedly assumed those of malignant pestilential fever. The day after this, the fourth, Mr. Mackenzie, justly alarmed, entreated me to see the patient, and the other carpenter who had been seized in a similar manner on the 24th. You may believe nothing effectual could now be done. Mr. Reid died on the fifth day, and the carpenter also on the fifth day of his fever. Here then, of four people, all of whom had been two or three years in the country, three died. I begged Mr. Mackenzie to employ every possible means to prevent the spreading of the evil from the manager's house, by burning every thing suspected of retaining infection, by fumigating with the nitric acid gas, and by white-washing. These directions I trust will be attended to, and have the desired effect.' This was the P. S. to my letter to Dr. Davidson, which either was not communicated to the Editors of the Med. Repository, or has been suppressed by them. It cannot, I apprehend, be denied, however, that it is an instance of disease proceeding immediately from contagion, '*the vitiated product of living vascular action,*' and mediately from infection, '*the venomous offspring of putrefaction:*' and we consequently see in it these two causes so subtilly distinguished, Med. Rep. v. 5, p. 186, acting, as they certainly always do, in succession, that is, as cause and effect, in the production of pestilential and typhus diseases. With small-pox and other specific contagions, as they are called, which are referred to "vitiated living vascular action," exclusively, although, observing the accustomed and undeviating course of nature, no doubt, originally the product of a peculiar infection, we have here nothing to do."

This history is more systematic than any we remember to have met with concerning the spreading of this destructive malady. It must, however, be remarked, that the four people, supposed to be immediately infected, and three of whom died, had been only two or three years in the country. It is well known the destructive seasons are uncertain, and frequently do not occur with violence for longer periods than the above mentioned. If it is really true, as the author seems to report on suspicion, that the disease, notwithstanding every precaution, spread to the dwelling house, such a fact would prove too much, as the yellow fever has never been suspected to be conveyed with such facility, and in spite of such precautions, by the greatest advocates for its contagious property. If there is really that analogy, which the author and Dr. M'Gregor contend for between plague and yellow fever, this degree of contagion would be still more improbable, as the last mentioned author does not admit that plague can be conveyed otherwise than by personal contact. We mean not to offer this remark as decisive on so important a subject, but merely to show, not only that the question remains with us undecided, but that we have not found sufficient arguments to induce us to alter the opinions we have hitherto maintained. We shall now refer to the correspondence contained in the Appendix. The first is a very long letter from Dr. Gordon, formerly of the Danish West India Island St. Croix, to Dr. Haygarth. This contains so much valuable luable matter, that we have made a minute examination of every part.

Dr. Gordon distinguishes this supposed contagion from the yellow fever, by the term Boulam, or, as he prefers calling it, the "pestilential fever." Its first appearance at St. Croix was from a Danish brig sent to St. Thomas to assist some vessels forced on shore by a violent south wind. The crew left St. Croix, eighty-four in number, all in perfect health, and after their return, only four were found to have escaped the disease.

"From the brig it was communicated rapidly to most of the European and North American vessels in the harbour, the crews of which were generally healthy before her return, and were more or less infected in proportion as they were more or less accustomed to the West India climate. On that account the crews of the droghers or coasting vessels were but partially infected.

"The soldiers in the garrison were in the same predicament. New comers were infected with the pestilential fever, while the old seasoned soldiers had only the common tropical remittents, and this was universally the case whenever both diseases were at the same time epidemic."

We are afterwards informed, that the disease raged violently till November, when the wind shifted suddenly to the North, and the weather was cooler; to this, says the author, "I attributed the sudden cessation of the influence of contagion." We confess, we should rather impute the cessation of a disease in such a manner, to a change in the constitution of the atmosphere.

"There was no further appearance of the disease until the month of July following, when a merchant ship, the Sovereign, Captain Story, from London, came into the harbour of Christianstadt. She had delivered goods at two islands in her passage; had in one of them received the infection, and lost in St. Croix three out of five in whom the disorder appeared.

"They were attended by another medical practitioner, but two ships that were lying, one on each side of her, were under my care, and in them I could evidently trace the origin and progress of the infection from the crews having intercourse with one another.

"From these ships the infection spread as in the former year, but the number of the infected, and of deaths, was less, as early application and other precautions were more generally attended to. I left the West Indies in September, 1794, and did not return until June, 1796. In my absence the pestilential fever had committed great ravages among the shipping.

"It appeared at various times in the hot months of 1796, 97, 98, and 99, and was principally introduced by American vessels, and those from St. Thomas, but more frequently by the former. We were on our guard against it, and whenever the fever appeared we traced it to its source, turned the vessel out of the harbour that brought it, cut off all communication of the infected with others, and if they had been brought on shore, or the disease any where appeared, took every precaution to prevent the further spreading of its malignant influence. Hence, though frequently imported, it was never general in the years above mentioned.

"In the year 1800, a pestilential fever of exactly the same nature, but rather with increased malignity, again appeared, and was communicated very generally to all who were susceptible of its contagion and came within the reach of its influence."

By these passages, it is evident that the contagion, if it was such, subsided till the return of the heats. As our author was absent part of the time, we will not inquire of him, why after the great success attending the means of prevention for four years, the same were not resorted to in the year 1800; however, as he returned in 1796, we might ask, whether there were any more precautions taken in 99 than in 1800? and, what is meant by the disease appearing in the last year with such increased violence as to attack all who were susceptible of the contagion, and came within its influence? It appears, that the first six months of that year were particularly healthy. After that time a mortal disorder attacked the new-comers, which was imputed to the marshy situation in which the cavalry barracks were built; and at first, the fever had a character to justify such a suspicion. At length it proved contagious. Several sick sailors were in the Hospital, and they communicated the disease to their comrades who visited them. "From them it spread among the shipping and inhabitants of the town, who were unaccustomed to the climate; for the Creoles, or those who were, as it is called, seasoned to the climate, were not affected by it."

Now, we confess ourselves at a loss to know, how this contagion was ascertained? If the fresh men who visited the hospital, caught the hospital fever, there would be nothing very extraordinary in such an event. But if a disease prevailed universally among all, excepting the Creoles, and if it began in an unhealthy spot, it must surely be difficult to ascertain, whether it was caught by mutual communication, or from the constitution of the atmosphere.

It is true, Dr. Gordon conceives, that he traced the disease to two recruits, who arrived in a convalescent state immediately from St. Thomas. But till we know how often the same arrivals happen without simliar consequences, and have also an explanation of the other events, we must be allowed to remain in doubt. Many other arguments follow, which we recommend to the perusal of such as are much interested in the question.

An Extract of a Letter follows from Dr. Haygarth to the College of Physicians at Philadelphia, dated October, 1806. This contains Dr. Fellows's account of the fever in Gibraltar, and its probable introduction from Cadiz by a person of the name of Sancho,

Sancho, who kept a retail grocer's shop in the heart of the town. This letter concludes in the following words:

"From numerous reports published by the anti-contagionists, I am clearly convinced that the sphere of infection in the pestilential fever, is confined to a very narrow distance, and that its progress might be effectually prevented by separation, cleanliness, and ventilation, exactly in the same manner as has been ever since 1783, successfully accomplished in regard to the typhus fever at Chester, and since 1796, at Manchester, and many other towns in Britain and Ireland."

If this opinion is right, we cannot help expressing our surprise at the rapid manner in which the disease spreads, and sometimes from causes which would appear inadequate to the effect. But still the question remains, is the disease at all contagious?

The next letter is from Dr. Robinson, dated College Square, Bristol. By this it appears, that the universal opinion in Gibraltar, was in favour of the contagious nature of the fever. Dr. Nooth's contrary opinion had indeed at first prevailed.

"However, in spite of this opinion," we are told, "some families and individuals secluded themselves from all communication with those attacked with the disease, and if they had not previous to their seclusion or segregation contracted the semina of infection, they experienced a happy exemption from this fever. Which was strikingly illustrated in the case of the family of Colonel Fyers, commanding engineer, a very intelligent gentleman, who with his numerous family secluded themselves in some retired part of the rock, and held no communication with the rest of the garrison or inhabitants. They all escaped the contagion, and are living monuments of his discernment and discretion.

"After the disease had abated, having exhausted itself by its own ravages partly, and the progress of infection being arrested by the approaching winter, as well as by the precautions that were ultimately adopted by authority, when the opinion of Dr. Nooth lost its influence."

There are two questions remain unanswered. Did those who shewed the disease after their seclusion infect the rest of their families? If the arrival of cold weather stopped the extent of the disease, is not such an event more conformable to a disease arising from the constitution of the air, than from contagion? Does such cold as the inhabitants of Gibraltar are accustomed to, ever stop the progress of small-pox?

The concluding Appendix, is an Extract from the log-book of the ship Hankey, by which it appears, that the crew were infected with ship fever, which spread according to the laws of that disease, but no way differently, as far as we can perceive.

*Observations*

*Observations on an Eruptive Disease which has lately occurred in the Town of Sherborne, in Dorset, after Vaccination, in a Letter to a Friend.* By Richard Pew, M. D. of Sherborne, Member of the R. M. S. and other Societies of Edinburgh.

Our great object in matters of controversy being to collect facts, and state them, if possible, in the author's language, we shall at once proceed to the only case of any importance; [the worst of all, to use our author's own words.]

"A delicate and pleasant countenanced boy, about six years old, who five years since was vaccinated by Mr. Gray, was a few weeks ago much frightened at the sight of a lad who had lost an eye, and was otherwise much disfigured by the small-pox, from which he was just recovered; some days after this he was seized with symptoms of fever, and, with a sort of intuitive presentiment, said, 'Mother, I am going to have the small-pox.' This was on Friday the twenty-third of September, and the pimples or rudiments of the future eruption were at this time, as Mr. Gray informs me, just distinguishable under the skin; in the night he was light-headed, and the next morning the pimples were considerably advanced; so that a general buzz prevailed over the town that this child had taken the *true* small-pox, after *true* and *constitutional* cow-pox. On Tuesday, the twenty-seventh, I happened to see him. The pustules (if, from their diminutive size, they might be so called,) bore so *general a resemblance* to real small-pox, that any one acquainted with the subject must immediately acknowledge them to be a *branch* of the *same family*; but he would immediately add, 'they are *much smaller*, *much less inflamed at the base*, *have a much* more *milky* appearance, and contain a much smaller quantity of matter than common small-pox usually do; they also seem likely to dry off much sooner, and to leave fewer marks or pits behind them, than is customary with common small-pox.' So mature, indeed, were the pustules on this (the fifth) day, that it was with some difficulty I could obtain matter enough from the face to inoculate one patient: I have been since informed, however, that effective matter was obtained from him so late as the seventh day; but I suppose this was taken from a part under the clothes; for parts which are covered do not dry off so soon as those which are exposed to the air. From the want of a subject to inoculate, the matter which I had taken was carried in my fob pocket for two days, in consequence of which (*as I suppose*) it failed to produce any effect; and in this respect it resembled the matter of cow-pox, for real small-pox matter is not, I believe, deprived of its infectious power by the heat of the human body.

"Three persons, however, were inoculated with *recent* matter, taken from this patient on the fifth day; a mother, and her child at the breast, in the workhouse; and a child, of about three years old, out of the workhouse. They all took the infection, the arms inflamed, inflamed, and each produced a *pustule*; but this pustule was, in my judgment, and in the judgment of Mr. Turner, a young and respectable surgeon, who is lately settled amongst us, very different from the *common small-pox pustule*; it was less elevated, was more widely diffused over the arm, was less inflamed at the base, and had that *blueish white tint*, something like skimmed or blue milk, which distinguishes the cow-pox pustule, instead of being of that *opaque cream-coloured white*, which, over a vermillion kind of base, distinguishes the small-pox pustule; in short, all the three pustules appeared to us to exhibit a sort of intermediate appearance between the small-pox and the cow-pox pustule; but as the surgeon who inoculated these patients did not perceive, or did not acknowledge that he perceived this difference, I must leave this fact (which I do without fear of contradiction) to the impartial decision of those who may repeat the experiment, which I am sorry I had it not in my power to do, from a want of matter, and from a want of a subject to operate upon."

The result of these inoculations was, that all three took the disease, and, as we have seen, had secondary eruptions, two of them few in number, and all passed through the complaint mildly, tho' no preparation or even subsequent regimen was much attended to. Many other practical remarks follow, which we omit, because they are not entirely new, particularly the remarks of the numerous advantages which cow-pox inoculation possesses over small-pox. In justice to the author's impartiality, however, we cannot but insert the following paragraph,

"No man of candour can, however, since the occurrence of these cases, continue to *urge* the practice of vaccination with that *implicit confidence* which he might have thought it his duty to do previously to their occurrence. And I cannot help being of opinion, that those medical gentlemen, who, from the most benevolent and disinterested motives (for they could have no other), have made a promise or vow never to inoculate for the small-pox, would be perfectly justified in departing from that vow, as

"*More honour'd*

"*I'the breach than the observance;*"

and after candidly stating all the advantages and disadvantages of small-pox and cow-pox inoculations, to leave parents unbiassedly to choose for themselves."

We meant to have finished after this, but the name of one of our employers caught our eye, and we must, like other fraternities, maintain the craft.

"From the compound nature of the disorder above spoken of, we learn, contrary to the opinion of the late ingenious Mr. John Hunter, a very curious fact, namely, that two distinct morbid* poisons

* I use the term morbid poisons as a phrase familiar to the faculty, on the authority of Mr. Hunter and Dr. Adams, and not from my own conviction of its propriety.

poisons may not only operate at the same time, on the same constitution, but that they may be communicated *together* to other individuals, and produce a compound disease. And from this fact we learn also a very important caution, namely, that parents, as well as surgeons, should be still more strictly upon their guard than they have heretofore been as to the health of those from whom they obtain the matter with which they inoculate; for if cow-pox matter, or the organic disposition to form it, can remain in the constitution for five or more years, as it did in this case, and form a compound with small-pox matter, which will operate on other constitutions, why may not the *fomes* or *materies morbi* of *syphilis*, *lepra*, &c. remain latent for an indefinite length of time, and be brought into action (as Mr. Hunter expresses it) by small-pox or cow-pox inoculations; and with the compound matter thus produced, communicate to other constitutions *scrophula*, *lepra*, *syphilis*, &c. Even the itch, it is said, may be communicated by small-pox inoculation, as the following fact, if it be a correct one, will evince:

"A friend of mine, the rector of a large parish, lately informed me, that a surgeon, who about eleven years ago inoculated the poor of his parish, to the amount of some hundreds, for the small-pox, took a considerable part of the matter he employed, from a family who had the itch, and that all the persons who were inoculated with this matter were affected with the itch, in addition to the small-pox. I do not mention these facts with a view to excite unnecessary alarm, but merely to guard surgeons and others against the *possibility* of making such communications, by always taking their small-pox matter from healthy persons, and, where practicable, from those *under fourteen* years of age."

We must first have better proofs of the compound nature of this disorder. We must next remark that these proofs are deficient, when we wish to assert that small-pox and cow-pox are different morbid poisons. According to Mr. Hunter's theory, they are the same, in as much as both will go through their actions at the same time, in the same person, which is not the case with two *different* morbid poisons. We wish this opinion of the sameness of the two diseases to be more generally understood, and if possible received. It has the sanction of the ingenious inventor of the vaccine practice, and if generally admitted, would do more to reconcile the public mind than all that can be written in favour of the well-known advantages of vaccination.

---

*Versuch einer Prufung und Verbesserung der jezt gewohnlichen Behandlungsart des Scharlach fiebers von* Dr. JOHANN HIEGLITZ. Hanover, 1 vol. 8vo.

THE Doctor begins with the assertion, that the scarlet fever is become more malignant and fatal than it formerly was, and that many authors are not at a loss to discover the cause, either in the seasons, sons, in the change of the atmosphere, or aliments, &c. not considering, that in other years, and in other countries, the same causes are prevalent, without the same effect, or often, the same effects are seen without the same cause.

Physicians, who reason unphilosophically, are ready at imputing the fatality of a disease to its peculiarity, to its complicated system, or to little inattentions, dietetic errors, passions of the patient, or catching cold; which last is often looked upon as an efficient cause, without any foundation, particularly as to the latter.

Our author expresses his doubts concerning the propriety of the common practice in scarlatina; he suspects that it is not only inadequate to the end, but even prejudicial, and productive of a fatal termination. He reasons with great judgment against the Brunonian system, and is surprized to find it adopted by physicians, who neither have read Brown, nor, he is apprehensive, any other medical book to any purpose.

He asserts, that the scarlet fever, in its beginning, is always of the sthenic kind; and that the inquiry, whether a fever is of a sthenic or asthenic nature, assists the practitioner much in the choice of the remedies.

If a disease is not opposed, as soon as it is formed, but allowed to run its own course through all its periods, and if the threatening symptoms are left to run their own course, then the physician is not master of his science, he has not courage to oppose disease, and should not boast that he has obtained the object of his wishes.

*Diaphoretics* and *external irritations* are in this disease *absolutely hurtful*. It is now an obsolete, and was never a proved idea, that morbid matter goes from the skin to the internal parts of the body. Even the most rigid humoral pathologists, least guided by any scientific knowledge, no more allow this theory.

To overcome all difficulty in the course of scarlatina, nothing is more efficacious than to combat and lower the fever immediately, and to give by this treatment a more favourable turn to the whole disease; if this is effected, the exanthema regulates itself favourably.

In exanthematic fevers every symptom originates certainly in the skin, and the future course of the distemper depends on the manner it begins. If the fever is perfectly formed, it is too late to produce any effect upon the exanthema by the way of the skin, and from this upon the fever. In regard to the fever, it is a question, whether the physician is to increase or to lower it, and what directions he ought to give to this purpose?

Unfortunately we are in the greatest ignorance, what quality of the skin be the fittest to a favourable direction of the exanthema, though the first and the most important turn of the disease depends on the state of this organ. However, the judgement is difficult. For instance, cleanliness is much favoured by bathing, and bodily cleanliness is conducive to health, yet dirt is no impediment to the powerful action of the skin, and does not hinder its important functions, functions, either in its healthy or morbid state. Common people, viz. day-labourers, peasants, &c. never wash their whole body, and very seldom their faces and hands, but perspire remarkably well, and their maladies end often with critical sweats.

It is very hurtful in the scarlatina, to direct the blood principally to the skin, and to force by this direction a perspiration, because the effect of it is a too great action of this organ, when a lessened one is more desirable; and later in the disease follows a too great a relaxation, when it is prejudicial.

The diaphoretics were formerly particularly recommended for the three following indications; which, however, all prove to be false.

1st. *To increase by the augmented perspiration of the skin, its secretion from the blood, and to depurate this the more.*

But there is no greater depuration of the blood wanted, because it is only the skin which originally suffers, and not the blood.

2d. *To fix by the perspiration the exanthema more to the skin, and to prevent by this, its turning inwards into the body.*

The exanthema has been often seen coming and going away, which gave the idea of its volatility and inconstancy, but this is commonly without any ill consequence. Sometimes it happens, that at the same time, danger of life and sufferings in the head and lungs have occurred; this gave rise to the hypothesis, that the exanthema went from the skin to the internal parts of the body. But the truth is, that the activity of the skin was exhausted by remedies given to increase the perspiration, and these deprived this organ of the energy necessary for the developement of the exanthema, rendering the skin as it were paralytic.

The skin is, after the scarlatina, even in those cases where little or no exanthema is observable, in such a state of debility and morbid irritability, that an accident of catching cold is sufficient to obstruct all perspiration, even so completely, that dropsy is the consequence. It was thought,

3d. *To prevent this inconvenience by promoting the perspiration.*

But only a relaxation and exhaustion of the skin, which render it more sensible to receive any contrary impression was effected. Physicians, consequently, precipitated their patients into evils, from which they intended to avert them, by their diaphoretic remedies.

After this follows *the plan of cure*, Dr. Hieglitz proposes as the most efficacious. He prescribes *emetics* and *purgatives* in the first period of illness, as very decisive remedies, after which cooling medicines, as neutral salts and acids, and a diet which corresponds with this treatment. The same treatment is often wanted throughout the whole course of the disease. In favour of this plan of cure, he pleads repeated and long experience.

At a time, when bile was accused as the cause of so many complaints, the epidemics of scarlatina were seldom ever malignant or very fatal; and, now the accounts of the increasing mortality are are elucidated by the history of it, it will be found, that it is parallel to the increasing influence of the Brunonian system in the same place. He also quotes, in favour of this plan of cure, the authority of an excellent practical physician, known likewise in the literary world, the late Dr. Wickmann, who cured this indisposition nearly in the same way as he here proposes. When the disease was very serious and obstinate, he used to prescribe the sulphuric acid. Our author adds, that the scarlet eruption, which sometimes appears on the skin, after having eaten muscles (mytilus edulis) is an additional proof that a scarlet exanthema can proceed from the bowels.

According to his opinion, no exanthematic fever ought to be called inflammatory, much less typhus, as the Brunonians used to call them always. Neither the one nor the other extreme is often found in nature. Is not the question, though without any practical use, of the most important influence in practice?

Emetics are powerful remedies not only to diminish but to cure sthenic fevers by the evacuation of the juices, which they effectuate, and by diminishing the irritability. Bleeding debilitates much more, and is therefore seldom indicated in these cases.

There are, however, neither sordes nor bile in the stomach and bowels, which call for the use of evacuants; nor does an accidental diarrhœa prove in favour of the evacuants, because it seldom happens to be critical, or of any salutary effect.

Measles, on the contrary, seldom allow the use of evacuants, and are, notwithstanding that, an exanthema, in which a critical diarrhœa is of the most beneficial consequence.

One symptom of the scarlatina directs distinctly our attention to the system of digestion, namely, the nausea and vomiting, which are observed at the beginning of the fever. Evacuants either re-establish the order of the affected parts of the abdomen, by which their re-action is lessened; or the fever affecting the bowels first, a beneficial influence may be gained over it, by acting upon them.

The physician is always more successful in each acute disease, if he well considers in the beginning what turn the disease is likely to take, either according to its own nature, or that of the epidemic. In an unfavourable course of the scarlatina, nothing is so common as affections of the brain; to prevent which, we can do nothing so powerfully (except putting epispastica to the extremities) as to administer evacuants, provided the patient is not in a state of too great debility. For, what is directed towards below and externally, cannot take its direction towards above and internally. The analogy, that evacuants are of decided use in great affections of the head, originating either from shocks, or apoplexy, or even in epilepsy, deriving the impetus of the blood from the brain, very much favours their employment in this illness.

Another

Another analogical proof of the utility of evacuants, is their success, when prescribed against erysipelas.

The Doctor speaks now against a publication, on the same subject, of Dr. Vogler, at Weilburg, which I pass over, as its contents will not interest the readers of this Journal. He continues to assert, that *single* symptoms must never attract too much the Physician's attention, but that his judgment must be drawn from the union of all the symptoms.

Among the emetics, he prefers the *ipecacuanha*, because it is certain in its operation, not having the effect of purgative medicines, like the antimonium tartarisatum. Its repetition is seldom wanted. When some hours after its effects are over, he advises laxative medicines, among which he recommends the *magnesia vitriolata*, dissolved in a sufficient quantity of water, and adding some oxymel simplex; he gives this till four evacuations follow; more, however, do no harm. For young children he prefers the aqua laxativa viennensis (a mixture of purgative medicines) to which manna alone, I think, is preferable. The diet of course must be as cooling as possible, viz. fruits, mucilaginous soups, and light vegetables; and for common drink, water, either alone or mixed with vinegar, acid of lemons, or vinegar of raspberries.

The same treatment ought to be continued two or three days, even if the disease does not immediately diminish in its violence, or if still serious symptoms appear, because he knows, as well from his own experience as from that of others, that this is the most successful treatment to shorten the fever, and to give a milder character to it in its progress, provided that no other symptoms require different remedies.

If the fever passes from the sthenic to the asthenic state, without other alarming symptoms, the ammoniacal muriat or the potus Riveri (saline draught) are sufficient remedies, together with clysters to open the body in case of costiveness, to see the disease end well. But if fever and heat increase, or the pulse augments; if the patient suffers more from restlessness, anguish, and inquietude, and if the delirium becomes more continued and grows worse, he then advises as a drink, sulphuric acid diluted with water and syrup; and omits this only, if symptoms appear which indicate an approaching hæmorrhagy of the nose. In which case he gives a grain of calomel, morning and evening, puts sinapisms to the extremities, principally to the feet; continues at the same time the ammoniacal muriat and the cooling diet, and is sure of good success by this treatment.

If the disease passes the third, fourth, fifth, or at a still later day, from the sthenic into the asthenic state, the weakening treatment must be omitted, and the diet changed to a more nourishing one. A not very strong infusion of *valeriana* with *liquor anodynus Hoffmanni*, or the *aqua ammoniaca acetata*, answers perfectly well, to bring the disease to a favourable end.

A higher

A higher degree of the fever requires more efficacious remedies, viz. An infusion of *serpentaria* with a large dose of *æther*; or a decoction of *bark*, if the asthenic state continues.

It will prove beneficial to use *sulphuric acid* diluted with water, as a common drink, if the circulation is very quick, and if the blood appears to cause congestions to the head, provided there are no spasms, which may demand a particular attention, and the state of the fever is not indirect asthenic.

He disapproves greatly of having immediate recourse to camphor, if scarlet fever should take a serious form; though it is given in small-pox and measles with the best effect. The only time to give it with success, is in the latter period of the disease, when those characteristic pains of the limbs make their appearance, which are always connected with suppressed cutaneous perspiration, and when the favourable end of the disease is pretty well ascertained.

*Opium*, if properly indicated, has an excellent effect, however it never can act as a principal; but only as an auxiliary remedy. Its proper time is when the symptoms are very spasmodic, and if the asthenic state has a duration and obstinacy, against which the common remedies are insufficient. Another proper time for its use is, when in the last period of the disease dubious diarrhœas appear.

*Moschus*, from 6 to 10 grains every four hours, is also an excellent remedy against nervous symptoms, especially against great anxiety and affections of the brain. Under the same circumstances, flowers of *arnica* are recommended, and repeated *sinapisms*, which are prefered to blisters.

The obstruction of the nose in the beginning of the disease is considered as a bad sign, being commonly united with affections of the head.

*Mercury* is advised as the best and most efficacious remedy in asthenic inflammations, especially in those of the brain and liver; also if the head is affected with delirium, sopor, or convulsions, four grains of calomel, or half a drachm of the mercurial ointment every day. It is often useful by its purging quality. All is gained, and the scene of sufferings is turned from the head, as soon as the salivary glands are affected by it, approaching to salivation. It often produces neither salivation nor purging, yet the most salutary effects follow. It is particularly well calculated for children, because it hardly ever causes in them a salivation. It is much recommended by Dr. Rush against thiscomplaint.

Dr. Hieglitz never saw the scarlet fever asthenic from the beginning; he remarks at the end of his treatise the three following particularities of the disease, which he thinks originate all from the same source.

1. *The animal heat rises in this illness, to a higher degree than in any other of our latitude, often to* 112° *Fahrenheit.*

*2. The pulse, as well in slight as malignant scarlet fevers, is more frequent than in any other disease.*

*3. The particular disposition to delirium.*

Some observations are added on Dr. Currie's method of cold affusion, which Dr. H. confesses never to have tried, because the method here explained satisfies him perfectly.

Such is the abstract of this valuable treatise, which seemed interesting enough to offer at this length to the readers of this Journal. They will regret with us the great difficulty, the almost impossibility, of procuring German publications on medical subjects.

